# Cardiac-Gated Diffusion-Weighted Magnetic Resonance Imaging Assessment of Kidney Function in Patients With Kidney Cancer

**DOI:** 10.1016/j.ekir.2026.106471

**Published:** 2026-03-11

**Authors:** Nima Gilani, Nalini Jeet, William C. Huang, Vasishta S. Tatapudi, Fang-Ming Deng, Kent Friedman, Karolina Soltys, Mary Bruno, Malika Kumbella, Michal L. Melamed, David M. Charytan, Xiaochun Li, Judith D. Goldberg, Artem Mikheev, Shavy Nagpal, Hersh Chandarana, Eric E. Sigmund

**Affiliations:** 1 Department of Radiology, Center for Advanced Imaging and Innovation (CAI^2^R), Center for Biomedical Imaging, NYU Langone Health, New York, New York, USA; 2 Department of Urology and Radiology, NYU Langone Health, New York, New York, USA; 3 Department of Medicine, NYU Langone Transplant Institute, NYU Langone Health, New York, New York, USA; 4 Department of Pathology, NYU Langone Health, New York, New York, USA; 5 Department of Radiology, NYU Langone Health, New York, New York, USA; 6 Department of Medicine, NYU Langone Chronic Kidney Disease Center, NYU Langone Health, New York, New York, USA; 7 Division of Nephrology, NYU Langone Health, New York, New York, USA; 8 Department of Population Health, NYU Langone Health, New York, New York, USA; 9 Department of Research Administration, NYC Health and Hospitals, New York, New York, USA

**Keywords:** cardiac phase, DTI, flow compensation, IVIM, kidney function, mGFR

## Abstract

**Introduction:**

Estimated glomerular filtration rate (GFR, eGFR) from serum creatinine outlines global kidney function, subject to biases in clinical scenarios from muscle wasting and inflammation (prevalent in kidney cancer) and failure to estimate split renal function (SRF, crucial in operative planning). Measured GFR (mGFR) (or true mGFR [mGFRt] without body surface area normalization) from diethylene-triamine-pentaacetate (^99m^Tc-DTPA) tracer clearance is the gold standard for bilateral kidney function, involving extended clearance times and radioactivity. Imaging-derived total kidney volumes are functional proxies but do not probe tissue quality.

**Methods:**

We employed advanced quantitative diffusion-weighted (DW) magnetic resonance (MR) imaging (MRI) at 3.0 T in addition to kidney volume measurements in a cohort of 27 patients with renal mass (26 and 18 underwent eGFR and mGFR tests, respectively). Cardiac-gated diffusion tensor imaging (DTI) and intravoxel incoherent motion (IVIM) parameters were derived. Individual MR metrics were evaluated for correlation with eGFR and mGFR with Pearson correlations and mixed-model analysis, respectively; LASSO-penalized multivariable regression was employed for mGFR prediction. The metrics were compared between proteinuria groups using 2-sample *t* tests.

**Results:**

Kidney volume correlated with renal function (split volume vs. split mGFR *r* = 0.54; split volume vs. split mGFRt *r* = 0.69). MR metrics correlated with individual kidney mGFR and mGFRt (*r* = 0.76 and 0.81, respectively). Mixed-effects LASSO multiple regression analysis predicted mGFR and mGFRt (R^2^ = 0.880 and 0.700, respectively). In addition, MR metrics differentiated proteinuria status.

**Discussion:**

Advanced DW MRI metrics may provide surrogates of mGFR and proteinuria. Parameters from bipolar encoding in diastole (emphasizing tubular flow) and flow compensation in systole (emphasizing vascular flow) were often informative.

## Graphical abstract

**Figure:**
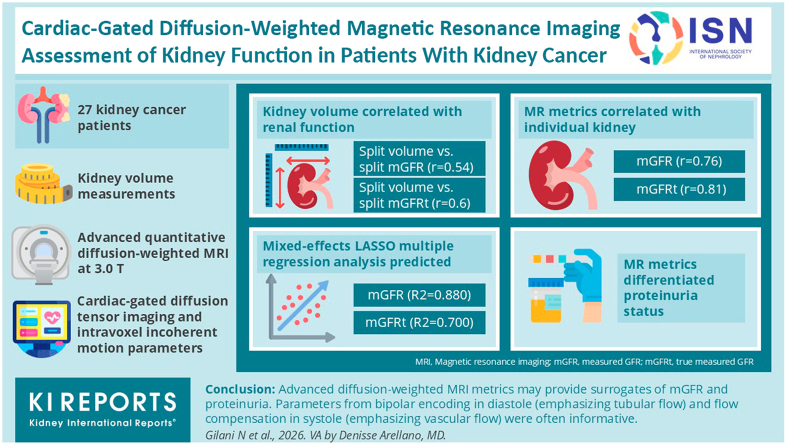

Accurate estimation of kidney function is crucial for managing patients who already have or are at risk of kidney disease.^1^ GFR is universally regarded as the best index of kidney function,^2^ and its estimation complements the characterization of kidney dysfunction in acute kidney injury and chronic kidney diseases such as diabetic kidney disease, polycystic kidney disease, or allograft rejection. In addition, it is important in the case of cancer and chemotherapy dosing.^3^ John and Pasha^4^ summarized the most commonly used markers for kidney function assessment, including urea, creatinine clearance, cystatin C, urine microscopy, urine osmolality, full blood count, electrolytes, and parathyroid hormone. Each of these metrics has application in the differential diagnosis of renal dysfunction. However, GFR is the starting point for patient management in this setting. GFR is either directly measured or indirectly estimated (mGFR and eGFR, respectively).^5^

eGFR, an estimate which is based on serum creatinine or cystatin-C levels and multivariable analytic models, is widely used in clinical practice. However, eGFR equations take clinical variables as surrogates for unmeasured physiologic processes such as creatinine generation (mostly from skeletal muscle), tubular secretion, reabsorption, and metabolism of filtration markers creatinine, and cystatin.^6^, ^7^, ^8^ Alternatively, it is possible to measure GFR by the injection of exogenous tracers, including radioactive-isotope–containing molecules such as ^99m^Tc-DTPA^9^ or ^125^I–iothalamate, and fitting a mathematical model to their washout curve from the body from blood or urine samples acquired at different time points after the injection.^10^ Radiography of these isotopes *in vivo* enables measurement of individual kidney mGFR (i.e., SRF).^11^ However, both eGFR and mGFR have limitations. In patients with renal masses, eGFR may be biased because of altered muscle mass and inflammation. Furthermore, it estimates overall GFR rather than lateralized measures of function in each kidney, which are crucial for operative planning (i.e., partial and radical nephrectomy) and predicting postoperative outcomes. mGFR is more accurate and can be lateralized but involves long scan times and ionizing radiation. Furthermore, GFR does not provide details of the mechanisms of function loss relevant for guiding intervention and management.

Numerous reports^12^, ^13^, ^14^, ^15^, ^16^, ^17^, ^18^, ^19^ recommend incorporation of MRI-derived parameters into clinical assessment of kidney function to stratify kidney diseases. MRI offers noninvasive assessment of a variety of tissue properties (oxygenation, metabolism, perfusion, microcirculation, and microstructure) with full spatial resolution of both kidneys, which is helpful in providing a more comprehensive assessment of kidney function than eGFR or mGFR alone. There are efforts to find the quantitative MR-derived parameters that are the most predictive measures of GFR.^20^ One example is diffusion-weighted imaging (DWI).

As an alternative to the first-order apparent diffusion coefficient representation of diffusion in tissue, the IVIM^21^ model of DW MR signal is highly relevant for kidney function assessment because of its separate estimates of diffusion and microcirculation of water molecules *in vivo*. More complex versions of IVIM^22^ have the potential to estimate blood flow velocity for special applications. In addition, because of the high microstructural anisotropy of the medullary pyramids within the kidney, diffusion tensor imaging (DTI)^23^ is widely used to investigate pathologies. Moreover, renal flow and microstructure anisotropy (REFMAP), inclusive of both DTI and IVIM, has the potential of outputting microcirculation- and microstructure-sensitive parameters^24^ (Figure 1). For the special case of kidney tissue, vascular and tubular contributions to water flow are both crucial to function, yet nontrivial to disambiguate. Therefore, to further increase the robustness of parameter estimates, REFMAP can employ a 4-quadrant acquisition strategy, including triggering the acquisitions at diastolic and systolic phases, as determined from the adjacent renal artery velocity,^25^ in addition to bipolar and flow-compensated MR gradient waveform pulse sequences^22^^,^^24^ (Figure 2). Research on methods such as REFMAP that are informative of independent biological features is warranted to characterize kidney function loss mechanisms. This includes testing the combined influence of gradient waveforms and cardiac phase encoding on MR assessments of kidney function, because both approaches modify the contributions of microcirculatory components. The variation of DWI contrast with these control variables may not only isolate more effective biomarkers but also increase their interpretability.

**Figure 1:**
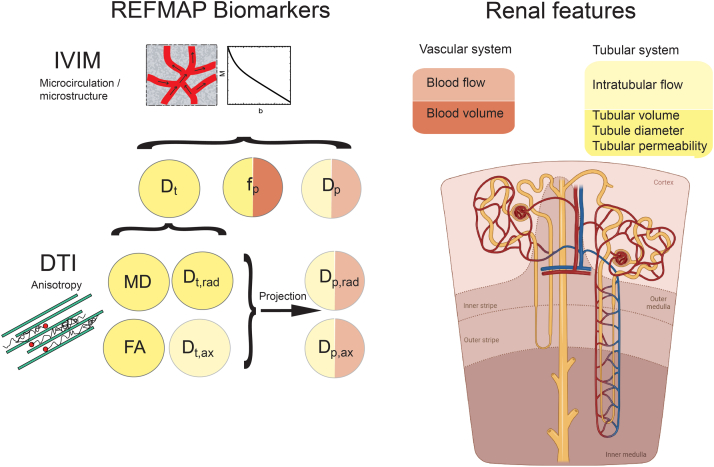
Diagram of REFMAP parameters and their hypothesized relationships with biophysical features of the nephron. (nephron diagram adopted from biorender.com with permission). D_p_, pseudodiffusivity; D_p,ax_, axial pseudodiffusivity; D_p,rad_, radial pseudodiffusivity; D_t_, diffusivity of water in the tissue; D_t,ax_, axial diffusivity of water in the tissue; D_t,rad_, radial diffusivity of water in the tissue; DTI, diffusion tensor imaging; FA, fractional anisotropy; IVIM, intravoxel incoherent motion; MD, mean diffusivity; REFMAP, REnal Flow and Microstructure AnisotroPy.

**Figure 2:**
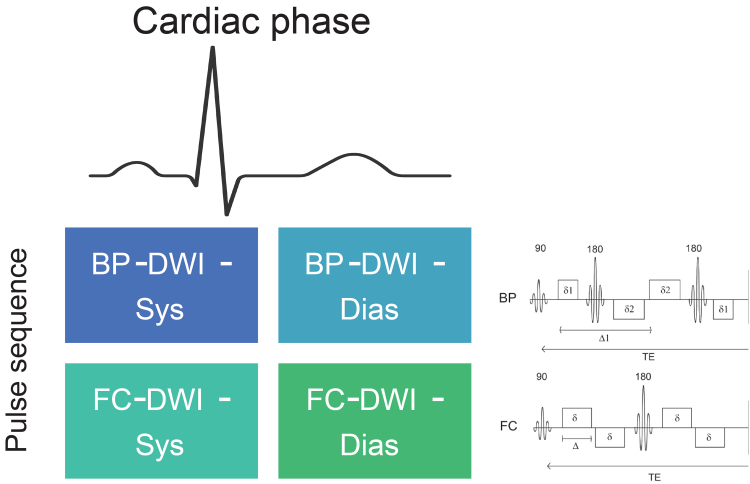
The 4-quadrant approach of renal flow and microstructure anisotropy, including 2 different DWI pulse and gradient sequences (right) and cardiac triggering at diastolic and systolic phases. BP, bipolar; Dias, diastole; DWI, diffusion-weighted imaging; FC, flow-compensated; sys, systole.

In addition, total kidney volume measured from MRI or computed tomography imaging has been correlated with kidney function, and remains one of the most clinically used and US Food and Drug Administration−approved methods in the context of polycystic kidney disease. Further, it has been recognized as a key cofactor in interpreting other kidney MR biomarkers such as T2 or T2∗.^26^

In this study, we evaluated the performance of noncontrast DWI REFMAP parameters, weighted by kidney volume, in estimating mGFR of individual kidneys in a cohort of patients with kidney cancer scheduled for partial nephrectomy. In addition, we compared these correlations with those of total kidney volume and eGFR as estimates of mGFR to better understand the potential added clinical value of REFMAP MRI. For this purpose, from a central kidney section, each of the REFMAP parameters was derived on a voxel-wise basis and averaged for that kidney in each tissue type (cortex and medulla). Further, each of these parameters was weighted by the total parenchymal volume of the corresponding kidney (i.e., left or right). This enabled a combination of kidney tissue quality (described from the perspective of each REFMAP parameter) and gross kidney volume. The REFMAP parameters (with and without kidney volume-weighting) were compared against their corresponding eGFR, ^99m^Tc–DTPA-based mGFR, and proteinuria status.

## Methods

### Subjects and Renal Function Measurements

In this Health Insurance Portability and Accountability Act–compliant and Institutional Review Board–approved prospective study, 27 patients with cancer (19 male/8 female, age: 61 ± 16 years, body mass index: 27.4 ± 5.4 kg/m^2^) provided written informed consent for participation in this study before partial nephrectomy. Standardized serum creatinine measurements were taken from bloodwork of 26 enrolled subjects to determine eGFR using the Chronic Kidney Disease-Epidemiology Collaboration 2021 equation. Proteinuria status was measured in 26 patients from urinalysis of spot urine collection dipstick measurement within ≤2 days before or after MR imaging. Eighteen of these patients elected to undergo ^99m^Tc-DTPA mGFR testing on the same day or ≤2 days before or after the MR imaging. ^99m^Tc-DTPA mGFR testing was performed similar to a previous study on cirrhotic patients^27^ as follows: One 22-gauge i.v. catheter was placed in each arm. After i.v. injection of 185 MBq of ^99m^Tc-DTPA and 20 ml of saline flush at 2 ml/s, coronal dynamic planar images were acquired over 8 minutes at 10-second intervals with a large field-of-view gamma camera (Symbia, Siemens) and low-energy collimator with 20% energy window centered at 140 keV, matrix 128 × 128 pixels, and pixel size 4.3 × 4.3 mm. At each of the 60- and 180-minute postinjection time points, contralateral to the initially injected arm, 2 blood draws were taken, each consisting of three 3 ml heparinized samples (the first one was discarded). The samples were centrifuged, and plasma activity was measured over 3 minutes in a well counter with background subtraction and correction for the ^99m^Tc decay. mGFR was determined using the plasma clearance of ^99m^Tc-DTPA. SRF was calculated by integrating the background-subtracted renograms over 2 to 3 minutes. Single kidney GFR was calculated by multiplying the mGFR by the SRF of that kidney. Supplementary Figure S1 shows the flowchart of patient recruitment and analysis, which includes cancer subtypes.

### MRI

Patients underwent abdominal imaging in a 3 T MRI system (MAGNETOM Prisma; Siemens Healthcare, Erlangen, Germany) in supine position with posterior spine array (4–6 elements activated) and anterior 18-channel body matrix array receiver RF coils, 4 electrocardiogram chest leads (Siemens Healthineers) for cardiac gating and scanner body coil RF transmission. The MR scan occurred on the same day as the renal function scan, except for 2 patients, where its occurrence was 1 day after and 2 days before. In Supplementary Table S1, we present a summary of pulse sequence parameters employed in this study. As part of the clinical routine, coronal oblique T2-weighted single shot fast spin echo (HASTE) images were collected for anatomical reference and parenchymal kidney volume measurements. Sagittal phase-contrast MRI images through the left renal artery were collected at multiple cardiac phases to estimate the systolic and diastolic cardiac phases for kidney tissue. A research application vendor-provided work-in-progress single-shot echo-planar imaging sequence with dynamic field correction was used to acquire bipolar and flow-compensated cardiac-triggered oblique coronal DW images aligned to the previous HASTE imaging. Other acquisition details were TR/TE 2800/81 ms, matrix 192/192/1, resolution 2.2/2.2/5 mm, GRAPPA acceleration factor 2, bandwidth 2170 Hz/pixel, and *b*-values of 0, 10, 30, 50, 70, 80, 100, 120, 200, 400, 600, and 800 s/mm^2^ in 12 directions. In addition, to correct for motion and field inhomogeneity, 16 right-to-left and 16 left-to-right phase-encoding *b* = 0 images were acquired, sampling the full range of motion for each kidney. The 4 DWI acquisitions (i.e., diastolic and systolic cardiac phases for each of bipolar and flow-compensated acquisitions) were each performed in 6 minutes, totaling 24 minutes. Flow-compensated acquisitions at the diastolic phase were not collected for 1 patient.

### Data Analysis

Parenchymal kidney volumes were segmented manually on the T2-weighted HASTE images because of their higher resolution than the DW images (the terms parenchymal and total kidney volumes are interchangeably used throughout the text). The volumes excluded lesions, cysts, the renal pelvis, and the renal artery or vein.

Marchenko Pasteur principal component analysis^28^ was performed for denoising on all DWI acquisitions. The kidneys were registered retrospectively to correct for breathing and cardiac motion (FireVoxel software). The left and right kidneys were processed independently to mitigate asynchronous left and right kidney motion and left-sided cardiac signal drop-out. To correct for field-inhomogeneity artifacts, the images were dewarped with FSL TOPUP,^29^ using the field map derived from images with reversed phase-encoding. This process took respiratory motion into account by applying a field map specific to each acquired image position. The processing flowchart was a replica of the one in Gilani *et al.*^30^ Image-by-image inspections were performed to exclude corrupted images for DTI and IVIM analysis.

The bipolar and flow-compensated DW images were processed using a custom code written in MATLAB and Statistics Toolbox (Release 2022a, The MathWorks, Inc., Natick, MA). First, average IVIM maps were generated from biexponential fits of the directionally averaged bipolar and flow-compensated DW image sets using the following equation:

(1.1) $$\frac{S}{S_{0}}=f_{p}\;\exp\left(-b\cdot D_{p}\right)+\left(1-f_{p}\right)\exp\left(-b\cdot D_{t}\right)$$

where *D*_*t*_ is defined as diffusivity of water in the tissue; *f*_*p*_ is the fraction of DWI signal that is affected by perfusion; and *D*_*p*_ is pseudodiffusion coefficient, which is sensitive to flow speed and architecture.^21^ *D*_*t*_ and *f*_*p*_ values were determined in a first fit to high *b*-values (*b* > 200 smm^−2^) and provided as first estimates. A second fit on all *b*-values with constrained *D*_*t*_ was performed to estimate *f*_*p*_ and *D*_*p*_. This approach is defined as segmented biexponential fit.

Second, the directional DWI signals from the acquisitions were processed analogously to the REFMAP approach^24^ to extract DTI, IVIM, and the following directional IVIM parameters: fractional anisotropy (*FA*), mean diffusivity (*MD*), axial diffusivity (*D*_tax_), radial diffusivity (*D*_trad_), scalar *f* with the same definition *as f*_p_, and mean (*D*_p_), axial (*D*_pax_), radial (*D*_prad_) pseudodiffusion coefficients. First, the *b*-value dependence of each voxel and direction was modeled by the segmented biexponential described earlier. The structural diffusivities (*D*_*t*_) were fit to a standard diffusion tensor model to derive *D*_tax_, *D*_trad_, and *MD* as well as *FA*.^31^^,^^32^ Next, at each voxel, pseudodiffusion coefficient (*D*_p_) was projected along the already derived axial and radial eigenvectors for *D*_t_ to derive its axial (*D*_pax_), radial (*D*_prad_), and mean (*D*_p_) versions. The final parameter set included DTI metrics (*MD*, *FA*, *D*_tax_ , and *D*_trad_) from the *D*_t_, as well as the scalar *f*, in addition to *D*_p_, *D*_pax_, and *D*_prad._

Cortex and medulla segmentations were performed using *b*0 images (cortical strip) and *FA* maps (medulla pyramid), from one of the DTI sets, and applied to the other series from that subject. Similar to the work by Gilani *et al.*^30^ and following consensus recommendations,^33^ these segmentations excluded nonparenchymal tissue (lesions, cysts, renal pelvis, and the renal artery or vein). A sample cortex-medulla segmentation is shown in Supplementary Figure S2. These regions of interest were used to collect mean values from all REFMAP parametric maps.

### Statistical Analysis

#### Renal Function Correlations

*eGFR versus mGFR*. Pearson correlation was calculated between estimated eGFR and mGFR values.

*Bilateral MR versus Bilateral mGFR*. Correlations were computed of MR biomarkers with renal function in the following 2 contexts: (i) MR biomarkers versus body surface area normalized mGFR (ml/min per 1.73 m^2^), and (ii) volume-weighted MR biomarkers (e.g. V∗*MD*) vs. “true” mGFRt (ml/min) without body surface area normalization.^34^ In both cases, bilateral kidney values (left and right) were separately considered along with bilateral mGFR values, derived from SRF percentages multiplied by mGFR. The correlation coefficient accounting for right and left side kidneys per subject is calculated using the mixed model approach proposed by Hamlett *et al.*,^35^ which explicitly defines the correlation structure within a subject’s repeated measurements. Correlations were estimated for each tissue/sequence/phase context separately (e.g. cortex/bipolar/systole; medulla/flow-compensated/diastole). Bilateral kidney volume was similarly correlated with split mGFRt. For all correlations, correlation coefficient, *r* and its standard error, 95% confidence interval (CI), and significance level, *P*, were determined (SAS software v 9.4).^36^ Adjusted *P*-values were provided for the multiple statistical comparison of the REFMAP versus mGFR/mGFRt correlations using the Benjamin-Hochberg method.

To consider all metrics of a given class (e.g., IVIM, DTI, or full REFMAP set, as well as their volume-weighted analogs) from different tissue/sequence/phase contexts for potential inclusion, mixed effects LASSO regression was used to generate multiparameter estimates of mGFR or (for volume-weighted metrics) mGFRt, that take within-subject correlations into account. The tuning parameter was selected using K-fold cross-validation, choosing the value that minimized the cross-validated deviance. The final LASSO-penalized generalized linear mixed model was then fitted using this optimal tuning parameter to obtain a parsimonious model accounting for random effects. We report selected metric coefficients and the *R*^*2*^ value for the predictions with mGFR (R software v 4.4, R Core Team, 2024).

*Average MR versus Average eGFR and mGFR*. Pearson correlations were computed from linear regression between left/right average MR biomarkers with eGFR. Correlations were estimated for each tissue/sequence/phase context separately (e.g., cortex/bipolar/systole, medulla/flow-compensated/diastole, etc.), as well as between eGFR and volume. For all correlations, the Pearson correlation coefficient (*r*) and the significance level (*P*) of its differing from 0 were determined. Multivariable regression was then performed by combining each average metric with eGFR for estimating mGFR. Regression coefficients were computed from bivariable regressions that include a single MR metric and eGFR versus mGFR. *P*-values are given reflecting whether the addition of that metric significantly improved prediction over that of eGFR alone.

#### Proteinuria

Each left/right average MR parameter in each context was compared between patients with and without proteinuria as determined by urinalysis using a 2-sample *t* test. The 2-sided significance level was set at 0.05.

## Results

Patient characteristics are summarized in Supplementary Table S2.

### Example MRI Maps

**Figure 3:**
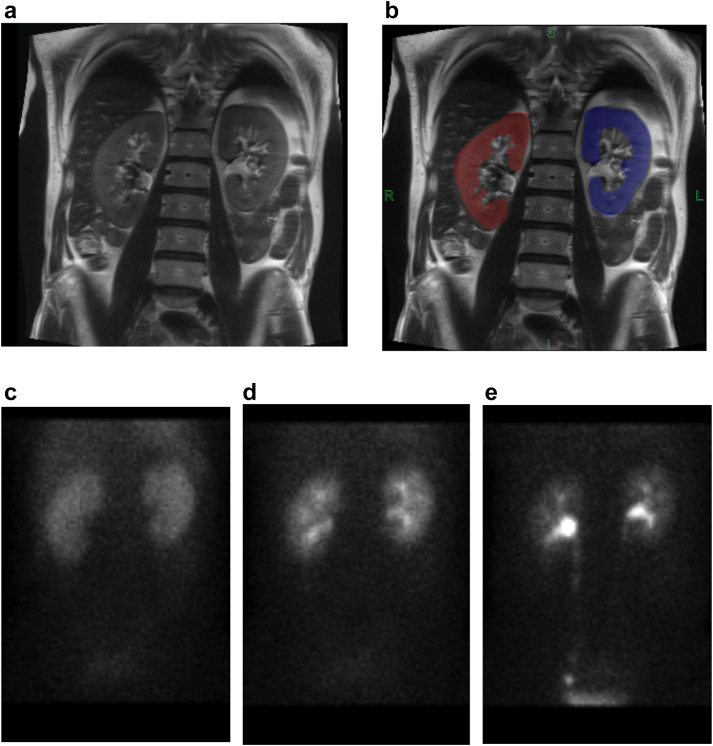
(a and b) Volume measurement from HASTE magnetic resonance imaging and (c) ^99m^Tc-DTPA dynamic planar scintigraphy (gamma camera) images for a renal mass patient in cortical, (d) medullary, and (e) urinary phases of clearance.

**Figure 4:**
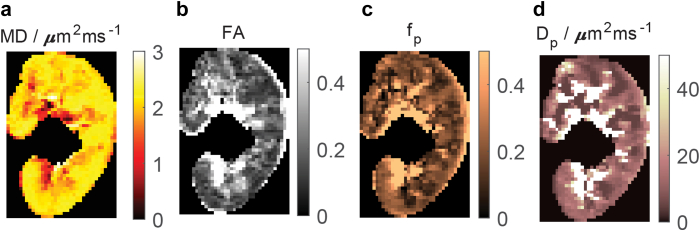
Renal flow and microstructure anisotropy parametric maps (a) MD, (b) FA, (c) f_p_, and (d) D_p_. D_p_, pseudodiffusivity; FA, fractional anisotropy; MD, mean diffusivity; f_p_, perfusion fraction.

In Figure 3, we show an example of kidney segmentation on HASTE MRI for volumetric measurement, and several gamma camera images of ^99m^Tc-DTPA over the course of its clearance (cortical, medullary, and urinary phases). In Figure 4, we show unilateral parametric map images (DTI and IVIM) within the REFMAP acquisition for the same patient.

### MRI-GFR Correlations

**Figure 5:**
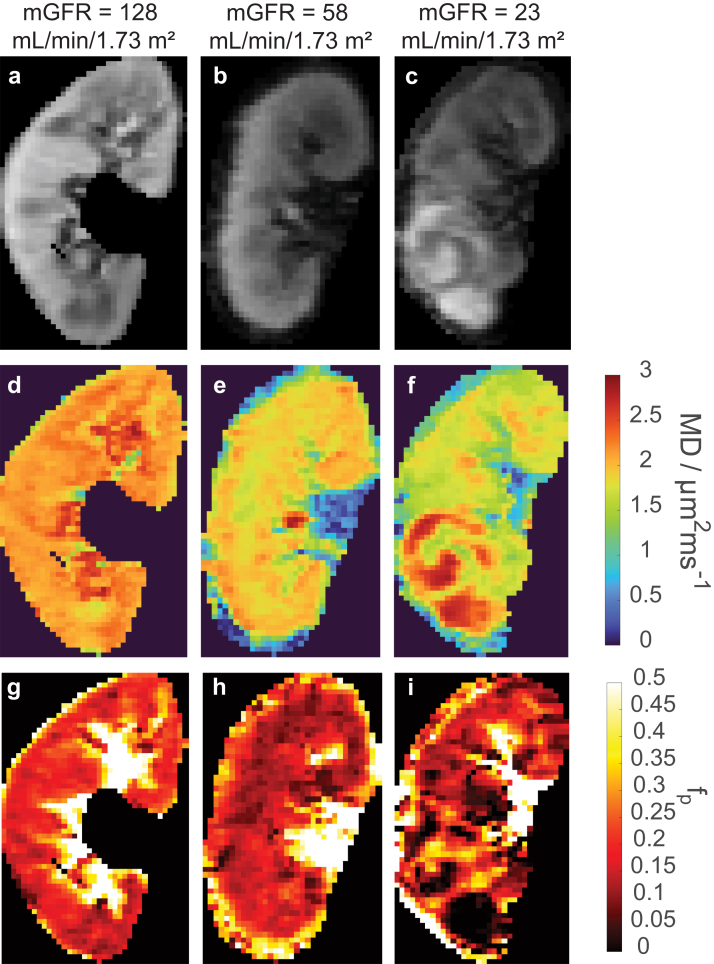
Example renal flow and microstructure anisotropy parametric maps from three patients with renal mass and with a range of mGFR values. (a–c) b0 images, (d–f) MD maps, (g–i) f_p_ maps are from bipolar diffusion-weighting in the diastolic phase. Both MD and f_p_ maps show clear trends of decreasing values and increasing heterogeneity with mGFR decline. f_p_, perfusion fraction; MD, mean diffusivity; mGFR, measured glomerular filtration rate.

In Figure 5, we show example MD and fp maps for 3 patients with varying mGFR values; both maps show a noticeable decline with decreasing mGFR. At the group level, there were high correlations between mGFR and eGFR values (*n* = 17) with *r* = 0.735 (95% CI: 0.394–0.898) and between mGFRt and average volume values (*n* = 18) with *r* = 0.724 (95% CI: 0.388–0.89). Similarly, split mGFR and mGFRt values significantly correlated with kidney volume (*r* = 0.549 [95% CI: 0.29–0.807] and *r* = 0.690 [95% CI: 0.50–0.878]), respectively.

**Figure 6:**
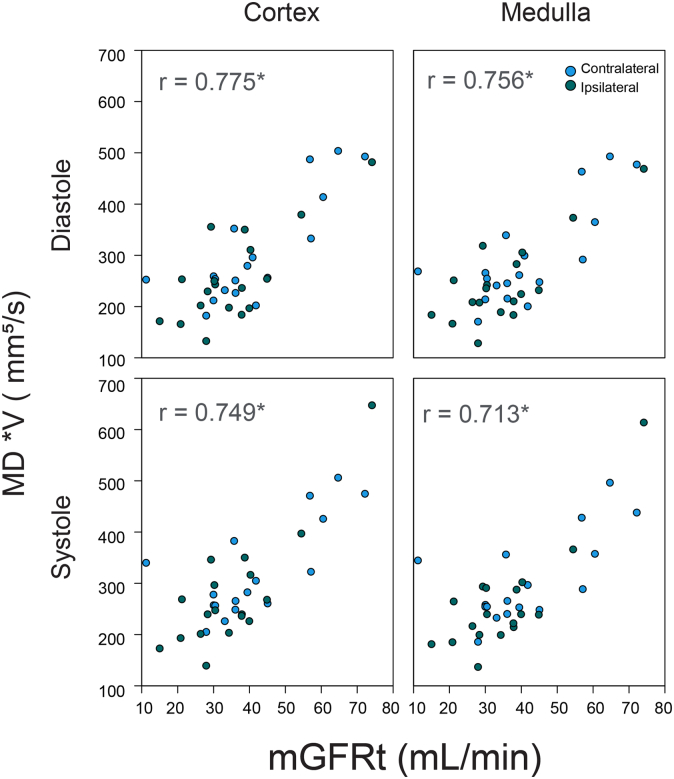
Plots of volume-weighted renal flow and microstructure anisotropy MD∗V (bipolar sequence) versus mGFRt. *r* is the correlation coefficient and ∗ indicates significance (adjusted p< 0.05). mGFRt, true measured glomerular filtration rate.

**Figure 7:**
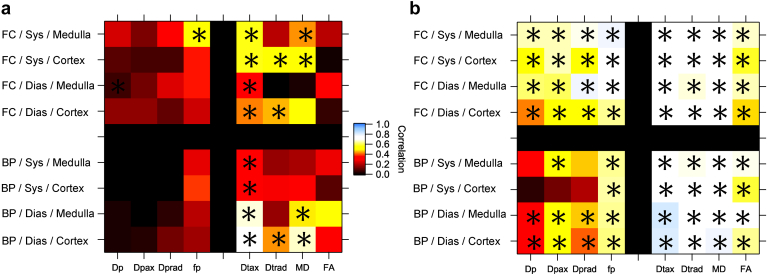
Heatmap comparisons of all the renal flow and microstructure anisotropy from the BP and FC pulse sequences, in the medulla and cortex, at the diastolic and systolic phases versus their corresponding (a) measured glomerular filtration rate (without volume-weighting), and (b) true measured glomerular filtration rate (with volume weighting). Md and Cx are medulla and cortex, respectively. ∗ indicates significant correlations (adjusted *P*-values < 0.05). Correlations are illustrated with a color scale of 0 < *r* < 1 to enable comparison between the original and volume-weighted correlations. As indicated in Table 3, only 8 of 64 contexts in Figure 6a (and none in Figure 6b) have correlations *r* < 0, none of which are significant. BP, bipolar; Dias, diastole; D_p_, pseudodiffusivity; D_p,ax_, axial pseudodiffusivity; D_p,rad_, radial pseudodiffusivity; D_t_, diffusivity of water in the tissue; D_t,ax_, axial diffusivity of water in the tissue; D_t,rad_, radial diffusivity of water in the tissue; FA, fractional anisotropy; FC, flow-compensated; f_p_, perfusion fraction; MD, mean diffusivity; sys, systole.

In Figure 6, we show example correlations of bilateral volume-weighted *MD* (V∗*MD*) with mGFRt values for bipolar encoding in both tissue types (cortex, medulla) and cardiac phases (systole, diastole). Significant correlations were found in all tissue-phase combinations between V∗*MD* and mGFRt. In Tables 1 and 2, and Supplementary Tables S3 and S4, we list all correlations, standard errors, 95% CIs, and significance levels for all parameter correlations, stratified by context. In Figure 7, we show heatmaps reflecting correlation coefficients between all REFMAP parameters (or volume-weighted analogs) in each tissue/sequence/phase context with mGFR (or mGFRt). On average, tissue diffusivities (*MD*, D_tax_, and D_trad_) show the highest correlation with mGFR, followed by perfusion fraction f_p_, and finally pseudodiffusivities (D_p_, D_pax_, and D_prad_). For perfusion fraction *f*_p_, the flow-compensated series in systole offers the strongest correlation with mGFR. Volume-weighted MR metrics showed stronger correlations with mGFRt than did unweighted MR metrics with mGFR. Maximal correlations of volume-weighted metrics for each context were derived from V∗D_tax_ (*r* = 0.77–0.81). The parameters providing significant and meaningful correlations with mGFR (*r* > 0.5, *P* < 0.05) originate exclusively from 2 “quadrants” of our DWI encoding scheme (Figure 2): bipolar / diastole (D_t_ in both cortex and medulla), and flow-compensated / systole (including both f_p_ and D_t_ in cortex and medulla). As will be discussed later, these quadrants may reflect sensitivity to tubular and vascular flow, respectively.

**Table 1:** Volume-weighted individual biomarker correlations with split mGFRt (bipolar sequence)

Metric	Tissue	Sequence	Phase	*R*	SE(*r*)	−95%	+95%	*P*	Adjusted *P*
D_t_	Cortex	Bipolar	Diastole	0.772^a^	0.073	0.628	0.915	< 0.001	< 0.001
D_p_	Cortex	Bipolar	Diastole	0.313	0.141	0.037	0.589	0.027	0.031
f_p_	Cortex	Bipolar	Diastole	0.627	0.108	0.415	0.838	< 0.001	< 0.001
MD	Cortex	Bipolar	Diastole	0.775^a^	0.072	0.633	0.917	< 0.001	< 0.001
FA	Cortex	Bipolar	Diastole	0.636	0.107	0.427	0.846	< 0.001	< 0.001
D_tax_	Cortex	Bipolar	Diastole	0.809^a^	0.062	0.687	0.930	< 0.001	< 0.001
D_trad_	Cortex	Bipolar	Diastole	0.730^a^	0.086	0.562	0.898	< 0.001	< 0.001
D_pax_	Cortex	Bipolar	Diastole	0.550	0.126	0.304	0.796	< 0.001	< 0.001
D_prad_	Cortex	Bipolar	Diastole	0.400	0.121	0.163	0.637	< 0.001	0.001
D_t_	Cortex	Bipolar	Systole	0.727^a^	0.081	0.568	0.885	< 0.001	< 0.001
D_p_	Cortex	Bipolar	Systole	0.054	0.178	−0.295	0.403	0.761	0.761
f_p_	Cortex	Bipolar	Systole	0.650	0.106	0.441	0.858	< 0.001	< 0.001
MD	Cortex	Bipolar	Systole	0.749^a^	0.077	0.598	0.899	< 0.001	< 0.001
FA	Cortex	Bipolar	Systole	0.587	0.118	0.356	0.819	< 0.001	< 0.001
D_tax_	Cortex	Bipolar	Systole	0.738^a^	0.080	0.581	0.894	< 0.001	< 0.001
D_trad_	Cortex	Bipolar	Systole	0.747^a^	0.077	0.595	0.898	< 0.001	< 0.001
D_pax_	Cortex	Bipolar	Systole	0.132	0.168	−0.198	0.462	0.432	0.458
D_prad_	Cortex	Bipolar	Systole	0.209	0.195	−0.173	0.592	0.284	0.310
D_t_	Medulla	Bipolar	Diastole	0.761^a^	0.077	0.610	0.913	< 0.001	< 0.001
D_p_	Medulla	Bipolar	Diastole	0.341	0.152	0.042	0.639	0.026	0.031
f_p_	Medulla	Bipolar	Diastole	0.639	0.103	0.438	0.840	< 0.001	< 0.001
MD	Medulla	Bipolar	Diastole	0.756^a^	0.079	0.602	0.911	< 0.001	< 0.001
FA	Medulla	Bipolar	Diastole	0.754^a^	0.079	0.600	0.908	< 0.001	< 0.001
D_tax_	Medulla	Bipolar	Diastole	0.813^a^	0.062	0.692	0.935	< 0.001	< 0.001
D_trad_	Medulla	Bipolar	Diastole	0.692^a^	0.096	0.503	0.880	< 0.001	< 0.001
D_pax_	Medulla	Bipolar	Diastole	0.490	0.134	0.228	0.753	< 0.001	< 0.001
D_prad_	Medulla	Bipolar	Diastole	0.464	0.148	0.175	0.753	0.002	0.002
D_t_	Medulla	Bipolar	Systole	0.706^a^	0.087	0.536	0.875	< 0.001	< 0.001
D_p_	Medulla	Bipolar	Systole	0.144	0.190	−0.228	0.516	0.448	0.460
f_p_	Medulla	Bipolar	Systole	0.602	0.113	0.381	0.824	< 0.001	< 0.001
MD	Medulla	Bipolar	Systole	0.714^a^	0.087	0.544	0.883	< 0.001	< 0.001
FA	Medulla	Bipolar	Systole	0.685	0.096	0.498	0.873	< 0.001	< 0.001
D_tax_	Medulla	Bipolar	Systole	0.739^a^	0.080	0.582	0.896	< 0.001	< 0.001
D_trad_	Medulla	Bipolar	Systole	0.682	0.094	0.499	0.866	< 0.001	< 0.001
D_pax_	Medulla	Bipolar	Systole	0.312	0.139	0.040	0.584	0.025	0.031
D_prad_	Medulla	Bipolar	Systole	0.279	0.171	−0.057	0.615	0.103	0.116

D_p_, pseudodiffusivity; D_pax_, axial pseudodiffusivity; D_prad_, radial pseudodiffusivity; D_t_, diffusivity of water in the tissue; D_tax_, axial diffusivity of water in the tissue; D_trad_, radial diffusivity of water in the tissue; FA, fractional anisotropy; MD, mean diffusivity; mGFRt, true measured glomerular filtration rate.

Significant correlations are indicated by *P* and adjusted *P* values < 0.05.

a Indicates significant correlations with larger correlation coefficients than that with split volume.

**Table 2:** Volume-weighted individual biomarker correlations with split mGFRt (flow-compensated sequence)

Metric	Tissue	Sequence	Phase	*R*	SE(*r*)	−95%	+95%	*P*	Adjusted *P*
D_t_	Cortex	FC	Diastole	0.744^a^	0.082	0.584	0.904	< 0.001	< 0.001
D_p_	Cortex	FC	Diastole	0.421	0.126	0.174	0.667	< 0.001	< 0.001
f_p_	Cortex	FC	Diastole	0.630	0.109	0.417	0.843	< 0.001	< 0.001
MD	Cortex	FC	Diastole	0.747^a^	0.081	0.588	0.905	< 0.001	< 0.001
FA	Cortex	FC	Diastole	0.476	0.134	0.213	0.738	< 0.001	< 0.001
D_tax_	Cortex	FC	Diastole	0.747^a^	0.080	0.591	0.904	< 0.001	< 0.001
D_trad_	Cortex	FC	Diastole	0.742^a^	0.083	0.580	0.904	< 0.001	< 0.001
D_pax_	Cortex	FC	Diastole	0.543	0.116	0.315	0.771	< 0.001	< 0.001
D_prad_	Cortex	FC	Diastole	0.533	0.128	0.283	0.784	< 0.001	< 0.001
D_t_	Cortex	FC	Systole	0.736^a^	0.084	0.572	0.900	< 0.001	< 0.001
D_p_	Cortex	FC	Systole	0.505	0.124	0.261	0.748	< 0.001	< 0.001
f_p_	Cortex	FC	Systole	0.701^a^	0.093	0.520	0.883	< 0.001	< 0.001
MD	Cortex	FC	Systole	0.737^a^	0.083	0.573	0.900	< 0.001	< 0.001
FA	Cortex	FC	Systole	0.576	0.122	0.337	0.815	< 0.001	< 0.001
D_tax_	Cortex	FC	Systole	0.733^a^	0.084	0.568	0.898	< 0.001	< 0.001
D_trad_	Cortex	FC	Systole	0.735^a^	0.084	0.571	0.900	< 0.001	< 0.001
D_pax_	Cortex	FC	Systole	0.659	0.093	0.476	0.843	< 0.001	< 0.001
D_prad_	Cortex	FC	Systole	0.550	0.120	0.315	0.785	< 0.001	< 0.001
D_t_	Medulla	FC	Diastole	0.703^a^	0.093	0.522	0.885	< 0.001	< 0.001
D_p_	Medulla	FC	Diastole	0.616	0.128	0.366	0.867	< 0.001	< 0.001
f_p_	Medulla	FC	Diastole	0.705^a^	0.094	0.521	0.888	< 0.001	< 0.001
MD	Medulla	FC	Diastole	0.701^a^	0.094	0.518	0.884	< 0.001	< 0.001
FA	Medulla	FC	Diastole	0.652	0.107	0.443	0.862	< 0.001	< 0.001
D_tax_	Medulla	FC	Diastole	0.729^a^	0.086	0.560	0.898	< 0.001	< 0.001
D_trad_	Medulla	FC	Diastole	0.671	0.101	0.472	0.869	< 0.001	< 0.001
D_pax_	Medulla	FC	Diastole	0.607	0.119	0.374	0.839	< 0.001	< 0.001
D_prad_	Medulla	FC	Diastole	0.780^a^	0.077	0.629	0.931	< 0.001	< 0.001
D_t_	Medulla	FC	Systole	0.738^a^	0.084	0.575	0.902	< 0.001	< 0.001
D_p_	Medulla	FC	Systole	0.656	0.079	0.501	0.810	< 0.001	< 0.001
f_p_	Medulla	FC	Systole	0.771^a^	0.077	0.620	0.922	< 0.001	< 0.001
MD	Medulla	FC	Systole	0.733^a^	0.085	0.567	0.900	< 0.001	< 0.001
FA	Medulla	FC	Systole	0.663	0.105	0.458	0.868	< 0.001	< 0.001
D_tax_	Medulla	FC	Systole	0.751^a^	0.080	0.594	0.909	< 0.001	< 0.001
D_trad_	Medulla	FC	Systole	0.712^a^	0.090	0.535	0.889	< 0.001	< 0.001
D_pax_	Medulla	FC	Systole	0.659	0.090	0.482	0.836	< 0.001	< 0.001
D_prad_	Medulla	FC	Systole	0.689	0.088	0.517	0.861	< 0.001	< 0.001

D_p_, pseudodiffusivity; D_pax_, axial pseudodiffusivity; D_prad_, radial pseudodiffusivity; D_t_, diffusivity of water in the tissue; D_tax_, axial diffusivity of water in the tissue; D_trad_, radial diffusivity of water in the tissue; FA, fractional anisotropy; FC, flow-compensated; f_p_, perfusion fraction; MD, mean diffusivity; mGFRt, true measured glomerular filtration rate.

Significant correlations are indicated by *P* and adjusted *P* values < 0.05.

a Indicates significant correlations with larger correlation coefficients than that with split volume.

From the LASSO-penalized multivariable regression prediction, of all parameter classes investigated, only IVIM parameters (D_t_, f_p_, and D_p_) and their volume-weighted sets provided convergent multivariate prediction. In Table 3, we show the coefficients for each parameter in each context, achieving an *R*^*2*^ = 0.880 for IVIM versus mGFR and *R*^*2*^ = 0.700 for volume-weighted IVIM versus mGFRt. Because the relationships of axial (D_pax_) and radial (D_prad_) pseudodiffusivity with mGFR were minimal, they were omitted from further correlation analyses.

**Table 3:** LASSO multivariable mixed effect regression between differently encoded renal IVIM parameters and mGFR

Parameter	Sequence	Tissue	Phase	Coefficients	Coefficients (w/volume weighting)
D_t_	Bipolar	Cortex	Diastole	2.841	0.894
D_p_	Bipolar	Cortex	Diastole	2.279	0.250
f_p_	Bipolar	Cortex	Diastole	0.000	0.791
D_t_	Bipolar	Cortex	Systole	1.132	0.788
D_p_	Bipolar	Cortex	Systole	−0.090	0.000
f_p_	Bipolar	Cortex	Systole	1.579	0.619
D_t_	Bipolar	Medulla	Diastole	3.211	0.871
D_p_	Bipolar	Medulla	Diastole	−2.960	−0.021
f_p_	Bipolar	Medulla	Diastole	1.651	0.447
D_t_	Bipolar	Medulla	Systole	0.303	0.755
D_p_	Bipolar	Medulla	Systole	−0.186	−0.244
f_p_	Bipolar	Medulla	Systole	−2.584	0.349
D_t_	FC	Cortex	Diastole	0.963	0.662
D_p_	FC	Cortex	Diastole	−0.364	0.334
f_p_	FC	Cortex	Diastole	−1.163	0.434
D_t_	FC	Cortex	Systole	0.802	0.598
D_p_	FC	Cortex	Systole	−1.297	1.060
f_p_	FC	Cortex	Systole	−0.130	0.700
D_t_	FC	Medulla	Diastole	−0.643	0.396
D_p_	FC	Medulla	Diastole	4.074	1.182
f_p_	FC	Medulla	Diastole	2.612	0.539
D_t_	FC	Medulla	Systole	2.081	0.571
D_p_	FC	Medulla	Systole	2.635	1.116
f_p_	FC	Medulla	Systole	2.457	0.866
Intercept				34.033	38.288
Tuning				0.604	9.567
*R^2^*				0.880	0.700

D_p_, pseudodiffusivity; D_t_, diffusivity of water in the tissue; FA, fractional anisotropy; FC, flow-compensated; f_p_, perfusion fraction; IVIM, intravoxel incoherent motion; MD, mean diffusivity; mGFR, measured glomerular filtration rate.

In Table 4, we show correlations of average MR parameters versus eGFR in each tissue/sequence/phase context. The most significant and meaningful (*r* > 0.5, *P* < 0.05) correlations were found for flow-compensated encoding in cortex. In Supplementary Tables S5 and S6, we show the correlations from multiple regression of average MR metrics / eGFR with mGFR, or MR metrics / volume / eGFR with mGFR, respectively. For several cortex metrics in diastole (D_t_, MD, and D_tax_ for bipolar sequence; D_t_ and D_tax_ for flow-compensated sequence), the combined mGFR prediction was significantly improved over that of eGFR versus mGFR alone. Similarly, cortical D_t_ and MD for bipolar sequence in diastole and systole provided significant improvement to mGFR correlation with a combination of eGFR and volume. This underlines the supplemental information on tissue quality provided by quantitative renal DWI.

**Table 4:** Correlation coefficients *r* and significance levels *P* of left/right average REFMAP parameters with estimated glomerular filtration rate

Bipolar								
	Cortex				Medulla			
	Diastole (*n* = 26)		Systole (*n* = 25)		Diastole (*n* = 26)		Systole (*n* = 25)	
	*r*	*P*	*r*	*P*	*r*	*P*	*r*	*P*
D_t_	0.533^a^	0.005^a^	0.058	0.783	0.415	0.035	0.045	0.831
D_p_	−0.188	0.358	−0.283	0.171	0.189	0.354	0.088	0.676
f_p_	0.016	0.938	0.257	0.214	0.366	0.066	0.248	0.232
MD	0.531^a^	0.005^a^	0.128	0.542	0.442	0.024	0.087	0.68
FA	0.155	0.449	0.041	0.845	0.362	0.069	0.273	0.187
D_tax_	0.469	0.016	0.136	0.517	0.644^a^	0^a^	0.22	0.292
D_trad_	0.363	0.068	0.11	0.601	0.124	0.545	−0.024	0.909


Flow-compensated								
	Cortex				Medulla			
	Diastole (*n* = 24)		Systole (*n* = 25)		Diastole (*n* = 24)		Systole (*n* = 25)	
	*r*	*P*	*r*	*P*	*r*	*P*	*r*	*P*
D_t_	0.486	0.016	0.578^a^	0.002^a^	0.319	0.129	0.18	0.389
D_p_	0.069	0.747	−0.19	0.363	0.047	0.827	−0.177	0.397
f_p_	0.089	0.679	0.126	0.55	0.29	0.169	0.336	0.1
MD	0.526^a^	0.008^a^	0.618^a^	0.001^a^	0.308	0.143	0.264	0.202
FA	−0.156	0.465	−0.384	0.058	0.132	0.539	0.061	0.772
D_tax_	0.421	0.04	0.481	0.015	0.459	0.024	0.328	0.11
D_trad_	0.529^a^	0.008^a^	0.661	0	0.172	0.422	0.181	0.386

D_p_, pseudodiffusivity; D_t_, diffusivity of water in the tissue; FA, fractional anisotropy; f_p_, perfusion fraction; MD, mean diffusivity; REFMAP, renal flow and microstructure anisotropy.

a Correlations with *r* > 0.5 and *P* < 0.05.

### MRI or Proteinuria Differentiation

In Table 5, we show group differences between patients with and without proteinuria in each tissue/sequence/phase context. Eight of the significant group differences occurred for the cortex in systole, involving both IVIM and DTI parameters. In each significant group difference, proteinuria-positive patients showed an average of 6.8% lower diffusivities, 38.6% lower perfusion fractions, 46.8% lower pseudodiffusivities, and 17.7% lower fractional anisotropies than proteinuria-negative cases.

**Table 5:** Comparison of left/right average REFMAP parameters with presence (Pos) or absence (Neg) of proteinuria in each context

*n*	Bipolar											
	Cortex						Medulla					
	Diastole			Systole			Diastole			Systole		
	Neg (22)	Pos (4)	*P*	Neg (21)	Pos (4)	*P*	Neg (22)	Pos (4)	*P*	Neg (21)	Pos (4)	*P*
D_t_	1.96 (0.09)	1.83 (0.12)	0.113	2.08 (0.24)^a^	1.97 (0.01)^a^	0.048^a^	1.89 (0.1)	1.81 (0.09)	0.193	1.98 (0.24)	1.94 (0.07)	0.533
D_p_	25.4 (10.28)	31.83 (15.47)	0.475	28.95 (15.36)	36.34 (21.34)	0.548	50.89 (20.26)	39.12 (26.3)	0.447	57.27 (27.84)	50.08 (23.66)	0.613
f_p_	0.16 (0.04)	0.13 (0.03)	0.127	0.21 (0.07)^a^	0.13 (0.05)^a^	0.044^a^	0.2 (0.05)	0.17 (0.02)	0.058	0.21 (0.06)^a^	0.15 (0.04)^a^	0.034^a^
MD	1.95 (0.1)	1.81 (0.13)	0.112	2.06 (0.21)^a^	1.96 (0.02)^a^	0.034^a^	1.88 (0.11)	1.81 (0.11)	0.262	1.95 (0.22)	1.93 (0.06)	0.732
FA	0.19 (0.07)	0.15 (0.05)	0.203	0.21 (0.05)	0.2 (0.02)	0.424	0.28 (0.06)	0.22 (0.05)	0.127	0.29 (0.05)^a^	0.25 (0.02)^a^	0.023^a^
D_tax_	2.34 (0.19)^a^	2.09 (0.16)^a^	0.045^a^	2.5 (0.3)	2.37 (0.05)	0.066	2.44 (0.18)^a^	2.22 (0.1)^a^	0.01^a^	2.53 (0.3)	2.45 (0.04)	0.225
D_trad_	1.76 (0.11)	1.67 (0.13)	0.272	1.85 (0.18)^a^	1.76 (0.03)^a^	0.046^a^	1.61 (0.12)	1.6 (0.13)	0.957	1.66 (0.19)	1.67 (0.07)	0.794


*n*	Flow-compensated											
	Cortex						Medulla					
	Diastole			Systole			Diastole			Systole		
	Neg (22)	Pos (4)	*P*	Neg (21)	Pos (4)	*P*	Neg (22)	Pos (4)	*P*	Neg (21)	Pos (4)	*P*
D_t_	1.90 (0.08)	1.86 (0.13)	0.405	2.05 (0.1)^a^	1.93 (0.06)^a^	0.026^a^	1.84 (0.08)	1.86 (0.13)	0.691	1.93 (0.08)	1.89 (0.05)	0.247
D_p_	34.25 (18.76)	43.12 (24.04)	0.526	31.74 (18.14)^a^	20.7 (3.34)^a^	0.017^a^	40.05 (22.1)	35.96 (28.75)	0.802	50.11 (24)^a^	29.59 (10.18)^a^	0.017^a^
f_p_	0.08 (0.02)	0.07 (0.03)	0.583	0.11 (0.04)	0.09 (0.01)	0.152	0.1 (0.03)^a^	0.07 (0.02)^a^	0.035^a^	0.11 (0.04)	0.09 (0.03)	0.158
MD	1.91 (0.08)	1.83 (0.13)	0.348	2.03 (0.1)^a^	1.9 (0.06)^a^	0.019^a^	1.8 (0.08)	1.83 (0.13)	0.664	1.89 (0.09)	1.86 (0.06)	0.543
FA	0.1 (0.03)	0.11 (0)	0.652	0.13 (0.03)	0.13 (0.03)	0.867	0.16 (0.05)^a^	0.13 (0.01)^a^	0.023	0.18 (0.04)^a^	0.15 (0.02)^a^	0.024^a^
D_tax_	2.1 (0.1)	2.02 (0.14)	0.352	2.28 (0.13)^a^	2.15 (0.03)^a^	0.001^a^	2.08 (0.09)	2.06 (0.15)	0.864	2.22 (0.11)	2.14 (0.07)	0.112
D_trad_	1.81 (0.09)	1.74 (0.12)	0.348	1.9 (0.1)	1.78 (0.09)	0.057	1.66 (0.11)	1.71 (0.11)	0.427	1.72 (0.1)	1.73 (0.06)	0.907

D_p_, pseudodiffusivity; D_t_, diffusivity of water in the tissue; FA, fractional anisotropy; f_p_, perfusion fraction; MD, mean diffusivity; REFMAP, renal flow and microstructure anisotropy.

Note: D_t_, D_p_, MD, Dtax, and Dtrad are in μm^2^ms^-1^.

a Significant differences (*P* < 0.05) between positive and negative groups are highlighted in bold.

## Discussion

The growing prevalence of MRI application to kidney function has been reviewed recently.^37^^,^^38^ In Table 6,^39^, ^40^, ^41^ we summarize recent studies correlating MR-derived quantitative DWI parameters with mGFR. There are numerous reports regarding the correlation of eGFR with DTI parameters (e.g., *r* = 0.35^42^ and 0.72^43^ for *FA*) or IVIM (e.g., *r* = 0.52,^44^ 0.45,^45^ and 0.46^46^ for IVIM perfusion fraction).

**Table 6:** Recent studies correlating DWI and volumetric MRI and mGFR in healthy controls, chronic kidney disease (CKD) and diabetic kidney disease (DKD)

Study	MRI parameter	Number of subjects and disease	Regression (r)	*P*-value
Li *et al.*^20^	ADC × renal parenchymal volume	36 patients with CKD and 12 healthy individuals	0.708	< 0.001
	ADC		0.493	< 0.001
	Renal parenchymal volume		0.337	0.018
Buchanan *et al.*^39^	Cortical ADC	22 volunteers with CKD and 22 healthy volunteers	0.717	0.02
Seah *et al.*^40^	Cortical ADC	32 volunteers with DKD and 10 healthy volunteers	0.6	< 0.001
	Medullary FA		0.3	< 0.001
Zhu *et al.*^41^	ADC	15 patients with CKD	0.546	0.002
	DKI		0.471	0.009
	SEM		0.227	0.228
	IVIM		0.789	< 0.001
	Combined		0.815	< 0.001

ADC, apparent diffusion coefficient; DKI, diffusion kurtosis imaging; FA, fractional anisotropy; IVIM, intravoxel incoherent motion; MRI, magnetic resonance imaging.

This study shows correlations between MR biomarkers and kidney function (mGFR) and proteinuria, both with and without consideration of kidney volume. Individually, the strongest correlations with kidney function were derived from the tissue compartment, especially *MD* and D_tax_, consistent with observations in the literature.^39^^,^^46^^,^^47^ These metrics were observed to be the only ones which in combination with eGFR, provided significantly improved estimation of mGFR than eGFR alone; previous studies have shown such a combination of diffusion MR metrics and eGFR to predict end-stage kidney disease.^48^ Mechanistically, higher axial tissue diffusivity may indicate higher tubular segment length, higher tubular volume, or minimal interstitial fibrosis in healthy kidney function. Furthermore, secondary correlations observed in perfusion fraction (consistent with the literature^45^^,^^46^) underline multiple mechanisms driving filtration (vascular flow in glomeruli, tubular integrity in loops of Henle) and suggest more comprehensive surrogate measures.

In addition, we observed variable mGFR correlations with diffusion gradient waveform (bipolar vs. flow-compensated) and cardiac phase (systole vs. diastole). At the acquisition level, almost all significant correlations emerged from 2 “quadrants”: bipolar / diastole (including D_t_ in both cortex and medulla), and flow-compensated / systole (including both f_p_ and D_t_ in cortex and medulla). In the case of LASSO regressions using all metrics from all of the eight (tissue/sequence/phase) contexts for mGFR estimation, the coefficients of metrics from these 2 quadrants were among the highest in the group as well. This analysis suggests that these 2 quadrants offer complementary markers of nephron function. Specifically, bipolar acquisition in diastole minimizes vascular flow but retains tubular flow and structure contributions, whereas flow-compensated acquisition in systole has opposite emphases (suppressing decay from slow tubular flow while maximizing fast vascular flow). In general terms, bipolar-diastolic acquisitions may address tubular pathologies (acute tubular necrosis and tubular atrophy), whereas flow-compensated systolic acquisitions may address vascular pathologies (glomerulosclerosis). In a related sense, when all parameters were included in a multivariable mixed effects LASSO regression model, performance achieved *R*^*2*^ of 0.880 and 0.700 for mGFR and mGFRt, respectively. These models highlight the advantage conferred by complementary MR feature combination and volume weighting in estimating kidney function. The results hint at how differently encoded DWI approaches can differentially sample and diagnose different components of the nephron and their modification by disease. Furthermore, such markers could be used to guide or monitor treatments of specific nephron components. Finally, as a future direction, by combining MRI biomarkers from multiple encodings and biophysical assumptions, it is possible to derive a novel modeling framework for kidney function, as has been shown in other organ contexts.^49^

The role of kidney size in kidney function is well-known. Sharma *et al.*^50^ showed a significant correlation between percentage volume and kidney function loss after partial nephrectomy. Courbebaisse *et al.*^11^ highlight the importance of reporting kidney function per parenchymal volume for kidney donors. Here, similar to a previous study,^20^ the influence of kidney size on function is significant, as shown by the increased correlations in Figures 6 and 7 and Table 3. In addition, volume-weighted versions of more specific MR biomarkers (D_tax_, D_tperp_, and *MD*) show higher correlation with true mGFR than kidney size, indicating the additive importance of tissue quality via diffusion MRI.

Regarding proteinuria, previous MRI studies have incorporated both proteinuria and MR diffusion metrics into multiple parameter models of renal function.^51^ However, less effort has focused on imaging markers of proteinuria. In this study, all parameters showing significant distinction show lower diffusivities or perfusion fractions in patients with proteinuria, and predominantly in the systolic phase in the cortex. These findings may be consistent with lower vascular flow, deterioration of glomerular barrier function,^52^^,^^53^ or higher viscosity filtrate, lowering average diffusion in the tubular space. The cardiac-gated systolic phase prevalence of significant differentiators seems to highlight a role for the vascular contribution to proteinuria in these patients.

Our study had some limitations. The sample size was small, which limited statistical power, particularly for the multivariable regression analysis. The pilot nature of this cohort implies that more evidence collection is warranted before generalization to other renal disease cohorts (chronic kidney disease, diabetic kidney disease, etc.). However, this issue is relevant for most multiparametric MR models of mGFR and may be further investigated with a higher sample size. Both mGFR and eGFR data were only available in a subset of the patients enrolled. Similarly, for the proteinuria outcome, a small number of cases were proteinuria positive. The cardiac-gated DWI acquisitions, though powerful in adding a range of encodings, were more time-consuming than ungated acquisitions, which increased overall scan time. It is warranted to test other diffusion MRI models for kidney function assessment, such as diffusion kurtosis imaging, because of sensitivity to kidney microstructure.^54^ In addition, because the total DWI scan time reached 24 minutes, it is required to design studies that select subsets of the 4 quadrants for specific disease imaging applications. We did not stratify analysis by any tumor properties (e.g., subtype or grade), nor did we consider longitudinal changes in function following nephrectomy or their prediction with MR metrics. This will be the subject of future work.

## Conclusion

In the present study, we have employed advanced diffusion MRI metrics, with variable gradient waveform and cardiac phase encoding, to produce surrogate models of kidney function and health as quantified by mGFR and proteinuria status. Individual or volume-weighted metrics, and multivariable combinations had model predictions of as high as *R*^*2*^ = 0.880 (multivariate model). Results suggest certain encoding combinations (bipolar waveform in diastole, flow-compensated waveform in systole) are particularly useful, possibly because of isolation of tubular and vascular components, and suggest a pathway for future streamlined acquisitions.

## Disclosure

HC reports grants from National Institutes of Health, during the conduct of the study; other funds from Siemens Healthineers, personal fees from Siemens Healthineers, and personal fees from United Imaging, outside the submitted work. DMC reports research grants and/or clinical trial contracts from the National Heart, Lung, and Blood Institute; the National Institute of Diabetes and Digestive and Kidney Diseases; AstraZeneca; Novo Nordisk; Eli Lilly/Boehringer Ingelheim; Vertex Pharmaceuticals; Medtronic; FifthEye; and Gilead. He receives royalties from UpToDate for editing review articles on hemodialysis-related topics. He has received consulting fees from Novo Nordisk, Boehringer Ingelheim–Eli Lilly, Merck, CSL Behring, Gilead Pharmaceuticals, LG Chem, GlaxoSmithKline, and Fresenius, and expert witness fees from the Proton Pump Litigation Consortium related to litigation regarding the role of proton pump inhibitors in chronic kidney disease progression. He has served on data safety monitoring boards or advisory boards for AstraZeneca, Alena Pharma, and LG Chem. All the other authors declared no competing interests.

## Supplementary Material

Supplementary File (PDF) **Figure S1.** Patient recruitment flowchart. **Figure****S2.** A sample cortex-medulla segmentation. **Table S1.** MR acquisition parameters of this study. **Table S2.** Summary of patient characteristics for this study. **Table S3.** Individual biomarker correlations with split mGFR (Bipolar sequence). **Table S4.** Individual biomarker correlations with split mGFR (flow-compensated sequence). **Table S5.** Correlation coefficients *R*^*2*^ and confidence intervals of a multiple regression of REFMAP parameters and estimated GFR (eGFR) to predict mGFR. **Table S6.** Correlation coefficients *R*^*2*^ and confidence intervals of a multiple regression of REFMAP parameters, total kidney volume, and estimated GFR (eGFR) to predict mGFR.

## Footnotes

Supplementary File (PDF)
**Figure S1.** Patient recruitment flowchart. **Figure** **S2.** A sample cortex-medulla segmentation. **Table S1.** MR acquisition parameters of this study. EPI: echo-planar imaging; DWI: diffusion-weighted imaging, PC-MRI: phase contrast MRI, T2w: T2-weighted, TR: repetition time, TE: echo time, venc: velocity encoding. **Table S2.** Summary of patient characteristics for this study. **Table S3.** Individual biomarker correlations with split mGFR (Bipolar sequence). **Table S4.** Individual biomarker correlations with split mGFR (flow-compensated sequence). **Table S5.** Correlation coefficients *R*^*2*^ and confidence intervals of a multiple regression of REFMAP parameters and estimated GFR (eGFR) to predict mGFR. *P*-values are given reflecting whether the addition of that metric significantly improved prediction over that of eGFR alone (significant cases in bold). **Table S6.** Correlation coefficients *R*^*2*^ and confidence intervals of a multiple regression of REFMAP parameters, total kidney volume, and estimated GFR (eGFR) to predict mGFR. *P*-values are given reflecting whether the addition of that metric significantly improved prediction over that of eGFR + volume alone (significant cases in bold).
