# Supplementary material for: Cardiac-Gated Diffusion-Weighted Magnetic Resonance Imaging Assessment of Kidney Function in Patients With Kidney Cancer
Source: Kidney Int Rep. 2026 Mar 11;11(5):106471. doi: 10.1016/j.ekir.2026.106471 (PMC13091829; doi:10.1016/j.ekir.2026.106471)

Supplemental File

Supplemental Table S1: MR acquisition parameters of this study. EPI: echo-planar imaging; DWI: diffusion-weighted imaging, PC-MRI: phase contrast MRI, T2w: T2-weighted, TR: repetition time, TE: echo time, venc: velocity encoding.

**Scan**	**TR/TE (ms)**	**Flip angle (º)**	**Matrix**	**Resolution (mm)**	**Orientation**	**Encodings**	**Notes**
T2w HASTE	1000/91 (effective)	120	320/320/20	1.1/1.1/5	Oblique coronal	-	-
PC-MRI	32.82/3.56	26	198/256/1	1.6/1.6/10	Oblique sagittal	24-27 phases	Venc: 80 cm/s
EPI-DWI	2800/81	90	192/192/1	2.2/2.2/5	Oblique coronal	b-values: 0, 10, 30, 50, 70, 80, 100, 120, 200, 400, 600, and 800 s/mm^2 <br>12 directions	1 Systolic, 1 diastolic cardiac phase <br>Bipolar / flow-compensated pulse sequences

Supplemental Table S2: Summary of patient characteristics for this study.

**Characteristic**	**Value**	**N**
Age (y)	61 ± 16	27
Gender	18 M / 9 F	27
Body mass index	27 ± 5.4	27
Kidney volume (cc)	290 ± 80	27
Proteinuria	22 (-) / 4 (+)	26
eGFR (ml/min/1.73 m^2)	79 ± 25	26
mGFR (ml/min/1.73 m^2)	69 ± 24	18
Renal mass subtype	Clear cell RCC	13
	Papillary RCC	6
	Chromophobe RCC	4
	Oncocytic renal neoplasm	2
	Fumarate hydratase deficient RCC	1

Supplemental Table S3: Individual biomarker correlations with split mGFR (Bipolar sequence). Significant correlations are indicated in bold. † indicates significant correlations with larger correlation coefficients than that with split volume.

**Metric**	**Tissue**	**Seq**	**Phase**	**r**	**SE(r)**	**-95%**	**+95%**	**p**	**Adj p**
D_t	Cortex	Bipolar	Diastole	-	-	-	-	-	-
D_p	Cortex	Bipolar	Diastole	0.0327	0.1607	-0.2823	0.3478	0.834	0.892
f_p	Cortex	Bipolar	Diastole	-	-	-	-	-	-
MD	Cortex	Bipolar	Diastole	**0.6631†**	0.0955	0.476	0.8503	**<0.001**	**<0.001**
FA	Cortex	Bipolar	Diastole	0.301	0.1638	-0.02	0.622	0.066	0.171
D_{tax}	Cortex	Bipolar	Diastole	**0.7585†**	0.0975	0.5674	0.9495	**<0.001**	**<0.001**
D_{trad}	Cortex	Bipolar	Diastole	**0.4315**	0.0944	0.2464	0.6165	**<0.001**	**<0.001**
D_{pax}	Cortex	Bipolar	Diastole	0.048	0.197	-0.3381	0.4342	0.807	0.892
D_{prad}	Cortex	Bipolar	Diastole	0.1335	0.1478	-0.1562	0.4232	0.366	0.598
D_t	Cortex	Bipolar	Systole	0.2956	0.1513	-0.001	0.5922	0.051	0.166
D_p	Cortex	Bipolar	Systole	-0.2376	0.1837	-0.5977	0.1225	0.196	0.434
f_p	Cortex	Bipolar	Systole	-	-	-	-	-	-
MD	Cortex	Bipolar	Systole	0.3024	0.158	-0.0073	0.612	0.056	0.166
FA	Cortex	Bipolar	Systole	0.1059	0.1827	-0.2522	0.464	0.562	0.762
D_{tax}	Cortex	Bipolar	Systole	**0.342**	0.0542	0.2359	0.4482	**<0.001**	**<0.001**
D_{trad}	Cortex	Bipolar	Systole	0.2878	0.1523	-0.0107	0.5863	0.059	0.166
D_{pax}	Cortex	Bipolar	Systole	-0.2133	0.1731	-0.5526	0.126	0.218	0.451
D_{prad}	Cortex	Bipolar	Systole	-0.1616	0.2044	-0.5622	0.239	0.429	0.665
D_t	Medulla	Bipolar	Diastole	-	-	-	-	-	-
D_p	Medulla	Bipolar	Diastole	0.0319	0.1856	-0.3319	0.3957	0.863	0.892
f_p	Medulla	Bipolar	Diastole	0.2117	0.1566	-0.0953	0.5187	0.176	0.421
MD	Medulla	Bipolar	Diastole	**0.4891**	0.1384	0.2179	0.7604	**<0.001**	**0.002**
FA	Medulla	Bipolar	Diastole	**0.502**	0.2315	0.0482	0.9558	**0.030**	0.117
D_{tax}	Medulla	Bipolar	Diastole	**0.6759†**	0.0943	0.4911	0.8607	**<0.001**	**<0.001**
D_{trad}	Medulla	Bipolar	Diastole	0.1745	0.1788	-0.176	0.5251	0.329	0.598
D_{pax}	Medulla	Bipolar	Diastole	-0.0589	0.1847	-0.421	0.3031	0.750	0.892
D_{prad}	Medulla	Bipolar	Diastole	0.0536	0.1987	-0.3359	0.4431	0.787	0.892
D_t	Medulla	Bipolar	Systole	0.3153	24.5097	-47.7238	48.354	0.990	0.990
D_p	Medulla	Bipolar	Systole	-0.1107	0.1926	-0.4882	0.2667	0.565	0.762
f_p	Medulla	Bipolar	Systole	-	-	-	-	-	-
MD	Medulla	Bipolar	Systole	0.1963	0.1669	-0.1309	0.5234	0.240	0.464
FA	Medulla	Bipolar	Systole	0.2729	0.738	-1.1736	1.7193	0.712	0.882
D_{tax}	Medulla	Bipolar	Systole	**0.3411**	0.1204	0.1051	0.5771	**0.005**	**0.020**
D_{trad}	Medulla	Bipolar	Systole	0.1702	0.1876	-0.1975	0.5379	0.364	0.598
D_{pax}	Medulla	Bipolar	Systole	-0.0922	0.1548	-0.3956	0.2111	0.551	0.762
D_{prad}	Medulla	Bipolar	Systole	-0.0908	0.1907	-0.4645	0.283	0.634	0.819

Supplemental Table S4: Individual biomarker correlations with split mGFR (Flow compensated sequence). Significant correlations are indicated in bold. † indicates significant correlations with larger correlation coefficients than that with split volume.

Metric	Tissue	Seq	Phase	r	SE(r)	-95%	+95%	p	Adj p
D_t	Cortex	FC	Diastole	**-**	-	-	-	**-**	**-**
D_p	Cortex	FC	Diastole	0.1696	0.1768	-0.177	0.5162	0.337	0.535
f_p	Cortex	FC	Diastole	**-**	-	-	-	**-**	**-**
MD	Cortex	FC	Diastole	**-**	-	-	-	**-**	**-**
FA	Cortex	FC	Diastole	**-**	-	-	-	**-**	**-**
D_{tax}	Cortex	FC	Diastole	**0.4257**	0.1377	0.1557	0.696	**0.002**	**0.008**
D_{trad}	Cortex	FC	Diastole	**0.4554**	0.1553	0.151	0.760	**0.003**	**0.012**
D_{pax}	Cortex	FC	Diastole	0.1626	0.1845	-0.199	0.524	0.378	0.550
D_{prad}	Cortex	FC	Diastole	0.1148	0.2113	-0.2993	0.529	0.587	0.731
D_t	Cortex	FC	Systole	**0.4973**	0.1384	0.226	0.7687	**<0.001**	**0.001**
D_p	Cortex	FC	Systole	0.1049	0.1968	-0.2807	0.4906	0.594	0.731
f_p	Cortex	FC	Systole	**0.3658**	0.1722	0.0282	0.7033	**0.034**	0.083
MD	Cortex	FC	Systole	**0.5121**	0.1355	0.2465	0.7778	**<0.001**	**0.001**
FA	Cortex	FC	Systole	0.0287	0.1937	-0.351	0.4085	0.882	0.882
D_{tax}	Cortex	FC	Systole	**0.5656†**	0.1248	0.3209	0.8103	**<0.001**	**<0.001**
D_{trad}	Cortex	FC	Systole	**0.5113**	0.1885	0.1418	0.8809	**0.007**	**0.019**
D_{pax}	Cortex	FC	Systole	0.0862	0.2122	-0.3296	0.502	0.684	0.744
D_{prad}	Cortex	FC	Systole	0.0856	0.2199	-0.3454	0.5165	0.697	0.744
D_t	Medulla	FC	Diastole	0.0775	0.1829	-0.2809	0.436	0.672	0.744
D_p	Medulla	FC	Diastole	0.2413	0.2391	-0.2273	0.71	0.313	0.527
f_p	Medulla	FC	Diastole	**0.3639**	0.1707	0.0294	0.6984	**0.033**	0.083
MD	Medulla	FC	Diastole	0.0383	0.1936	-0.3412	0.4178	0.843	0.870
FA	Medulla	FC	Diastole	0.2918	0.1756	-0.0523	0.6359	0.097	0.221
D_{tax}	Medulla	FC	Diastole	**0.345**	0.0868	0.1749	0.5151	**<0.001**	**<0.001**
D_{trad}	Medulla	FC	Diastole	-0.0886	0.095	-0.2748	0.0976	0.351	0.535
D_{pax}	Medulla	FC	Diastole	0.133	0.2676	-0.3915	0.6576	0.619	0.734
D_{prad}	Medulla	FC	Diastole	0.2586	0.2223	-0.1772	0.6943	0.245	0.461
D_t	Medulla	FC	Systole	**0.4081**	0.0753	0.2605	0.5556	**<0.001**	**<0.001**
D_p	Medulla	FC	Systole	0.248	0.1724	-0.0899	0.5858	0.150	0.301
f_p	Medulla	FC	Systole	**0.5431**	0.1427	0.2635	0.8228	**<0.001**	**<0.001**
MD	Medulla	FC	Systole	**0.4347**	0.1593	0.1225	0.7469	**0.006**	**0.019**
FA	Medulla	FC	Systole	0.2138	0.365	-0.5016	0.9293	0.558	0.731
D_{tax}	Medulla	FC	Systole	**0.5666†**	0.124	0.3235	0.8098	**<0.001**	**<0.001**
D_{trad}	Medulla	FC	Systole	0.2144	0.1923	-0.1625	0.5914	0.265	0.471
D_{pax}	Medulla	FC	Systole	0.1452	0.1796	-0.2069	0.4973	0.419	0.583
D_{prad}	Medulla	FC	Systole	0.2907	0.1891	-0.0799	0.6613	0.124	0.265

Supplemental Table S5: Correlation coefficients R^2 of a multiple regression of REFMAP parameters and estimated GFR (eGFR) to predict mGFR. P-values are given reflecting whether the addition of that metric significantly improved prediction over that of eGFR alone (significant cases in bold).

Bipolar								
	Cortex				Medulla			
	Diastole (N=17)		Systole (N=16)		Diastole (N=17)		Systole (N=16)	
	R^2	p	R^2	p	R^2	p	R^2	p
D_t	0.78(0.429,0.904)	**0.002**	0.615(0.152,0.825)	0.138	0.594(0.147,0.809)	0.195	0.571(0.104,0.801)	0.357
D_p	0.545(0.096,0.781)	0.732	0.541(0.076,0.784)	0.905	0.567(0.118,0.794)	0.374	0.543(0.077,0.785)	0.823
f_p	0.585(0.137,0.804)	0.242	0.541(0.076,0.784)	0.979	0.544(0.096,0.781)	0.747	0.551(0.085,0.79)	0.6
MD	0.745(0.365,0.887)	**0.005**	0.612(0.149,0.823)	0.146	0.568(0.12,0.795)	0.36	0.557(0.091,0.793)	0.498
FA	0.596(0.15,0.81)	0.187	0.555(0.088,0.792)	0.537	0.581(0.132,0.802)	0.268	0.553(0.087,0.791)	0.556
D_{tax}	0.732(0.342,0.881)	**0.007**	0.608(0.144,0.821)	0.158	0.63(0.19,0.829)	0.087	0.563(0.096,0.796)	0.434
D_{trad}	0.582(0.133,0.802)	0.261	0.605(0.14,0.82)	0.17	0.542(0.094,0.78)	0.832	0.552(0.086,0.79)	0.576
Flow Comp								
	Cortex				Medulla			
	Diastole (N=16)		Systole (N=17)		Diastole (N=16)		Systole (N=17)	
	R^2	**p**	R^2	**p**	R^2	p	R^2	p
D_t	0.665(0.216,0.851)	**0.047**	0.648(0.214,0.838)	0.057	0.542(0.076,0.784)	0.888	0.582(0.134,0.802)	0.259
D_p	0.542(0.077,0.785)	0.853	0.556(0.107,0.788)	0.499	0.551(0.085,0.79)	0.592	0.607(0.162,0.816)	0.145
f_p	0.545(0.08,0.786)	0.731	0.612(0.168,0.819)	0.131	0.557(0.091,0.793)	0.501	0.647(0.213,0.838)	0.059
MD	0.635(0.176,0.835)	0.09	0.62(0.178,0.823)	0.108	0.543(0.078,0.785)	0.807	0.559(0.11,0.789)	0.454
FA	0.583(0.117,0.808)	0.269	0.543(0.095,0.78)	0.791	0.588(0.122,0.811)	0.242	0.541(0.093,0.779)	0.942
D_{tax}	0.677(0.233,0.857)	**0.036**	0.623(0.181,0.825)	0.103	0.566(0.099,0.798)	0.402	0.564(0.115,0.792)	0.405
D_{trad}	0.589(0.122,0.811)	0.24	0.607(0.161,0.816)	0.148	0.557(0.09,0.793)	0.509	0.552(0.103,0.785)	0.567

Supplemental Table S6: Correlation coefficients R^2 and confidence intervals of a multiple regression of REFMAP parameters, kidney volume, and estimated GFR (eGFR) to predict mGFR. P-values are given reflecting whether the addition of that metric significantly improved prediction over that of eGFR+volume alone (significant cases in bold).

Bipolar								
	Cortex				Medulla			
	Diastole (N=17)		Systole (N=16)		Diastole (N=17)		Systole (N=16)	
	R^2	p	R^2	p	R^2	p	R^2	p
D_t	0.789(0.397,0.902)	**0.003**	0.708(0.216,0.863)	**0.04**	0.66(0.169,0.833)	0.103	0.689(0.185,0.852)	0.063
D_p	0.582(0.073,0.788)	0.792	0.58(0.047,0.791)	0.926	0.598(0.091,0.798)	0.451	0.581(0.048,0.791)	0.858
f_p	0.611(0.106,0.805)	0.322	0.581(0.048,0.791)	0.849	0.58(0.071,0.787)	0.878	0.593(0.061,0.799)	0.541
MD	0.758(0.334,0.886)	**0.008**	0.712(0.222,0.865)	**0.037**	0.632(0.132,0.818)	0.195	0.667(0.153,0.841)	0.101
FA	0.627(0.125,0.814)	0.222	0.585(0.052,0.794)	0.716	0.615(0.11,0.808)	0.294	0.586(0.053,0.794)	0.673
D_{tax}	0.748(0.314,0.881)	**0.011**	0.679(0.17,0.847)	0.078	0.727(0.275,0.87)	**0.02**	0.667(0.153,0.841)	0.101
D_{trad}	0.613(0.108,0.807)	0.305	0.722(0.239,0.87)	**0.029**	0.585(0.076,0.79)	0.693	0.652(0.132,0.832)	0.14
Flow Comp								
	Cortex				Medulla			
	Diastole (N=16)		Systole (N=17)		Diastole (N=16)		Systole (N=17)	
	R^2	p	R^2	p	R^2	p	R^2	p
D_t	0.684(0.178,0.85)	0.069	0.672(0.187,0.84)	0.078	0.588(0.055,0.796)	0.634	0.635(0.135,0.819)	0.185
D_p	0.582(0.049,0.792)	0.798	0.599(0.092,0.798)	0.437	0.612(0.081,0.81)	0.339	0.63(0.129,0.816)	0.206
f_p	0.581(0.048,0.792)	0.833	0.619(0.115,0.81)	0.267	0.583(0.05,0.792)	0.778	0.649(0.154,0.827)	0.133
MD	0.665(0.15,0.84)	0.106	0.645(0.149,0.825)	0.144	0.581(0.048,0.792)	0.836	0.61(0.104,0.805)	0.331
FA	0.601(0.069,0.803)	0.439	0.579(0.07,0.786)	0.986	0.599(0.067,0.802)	0.467	0.581(0.072,0.787)	0.853
D_{tax}	0.69(0.187,0.853)	0.061	0.638(0.139,0.821)	0.172	0.606(0.075,0.806)	0.389	0.604(0.097,0.801)	0.388
D_{trad}	0.63(0.103,0.82)	0.225	0.641(0.143,0.823)	0.16	0.581(0.048,0.791)	0.862	0.603(0.096,0.8)	0.398

Supplemental Figure S1: Patient recruitment flowchart.

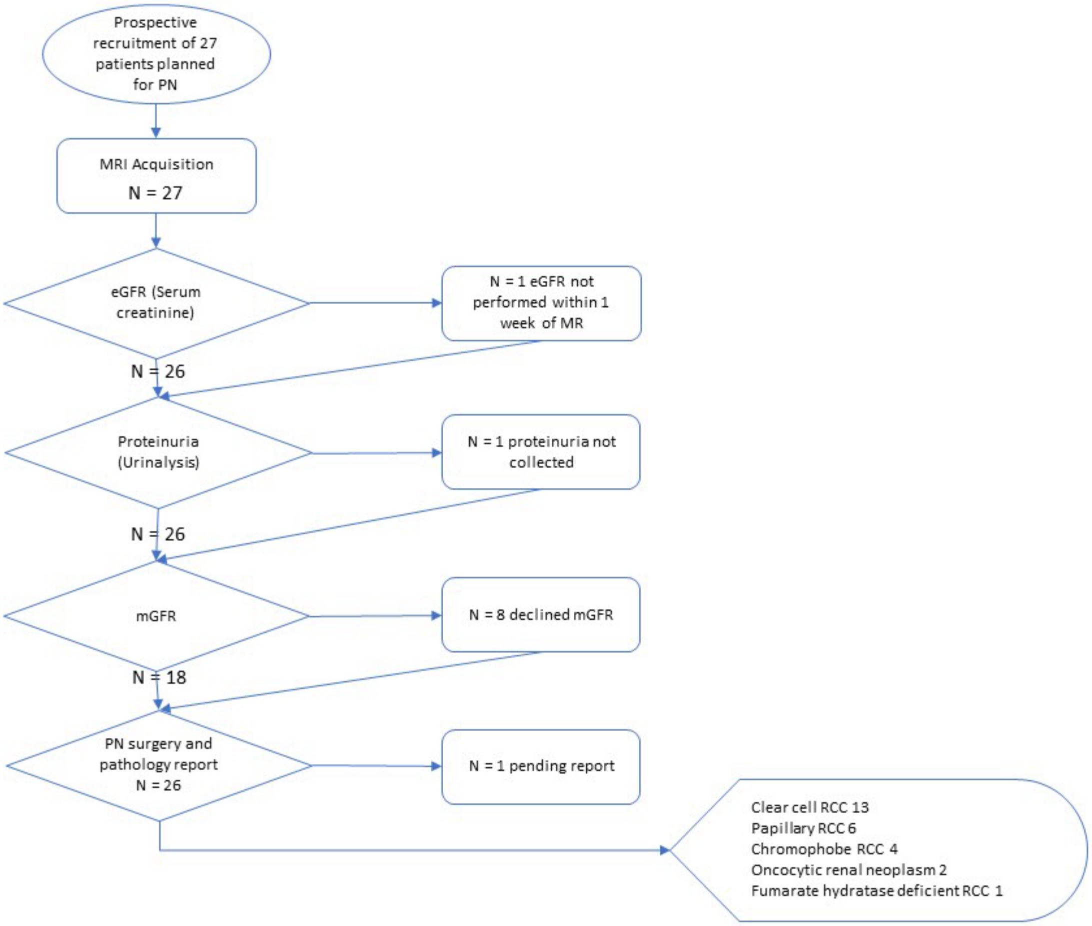

Supplemental Figure S2: A sample cortex-medulla segmentation.

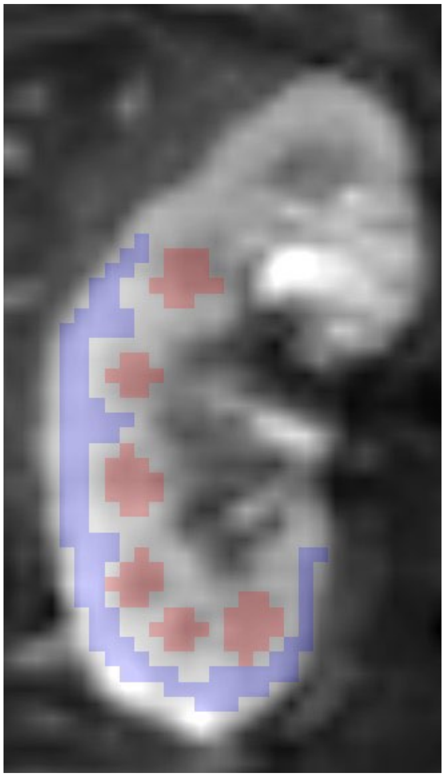

Supplement: Supplementary File (PDF) — Figure S1. Patient recruitment flowchart. FigureS2. A sample cortex-medulla segmentation. Table S1. MR acquisition parameters of this study. Table S2. Summary of patient characteristics for this study. Table S3. Individual biomarker correlations with split mGFR (Bipolar sequence). Table S4. Individual biomarker correlations with split mGFR (flow-compensated sequence). Table S5. Correlation coefficients R2 and confidence intervals of a multiple regression of REFMAP parameters and estimated GFR (eGFR) to predict mGFR. Table S6. Correlation coefficients R2 and confidence intervals of a multiple regression of REFMAP parameters, total kidney volume, and estimated GFR (eGFR) to predict mGFR. [file mmc1.pdf]
